# GABAergic neurons in the nucleus accumbens regulate hedonic food intake via orexin-A expression in the lateral hypothalamus

**DOI:** 10.22038/ijbms.2021.58140.12923

**Published:** 2021-09

**Authors:** Jieting Kong, Shengli Gao, Xiaoman He, Nana Zhang, Jinfang Huang, Pengfei Ji, Shouheng Yao, Xiang Ren, Yiru Wang, Yanling Gong, Feifei Guo

**Affiliations:** 1 Department of Physiology and Pathophysiology, School of Basic Medicine, Qingdao University, Qingdao, China; 2 Department of Special Medicine, School of Basic Medicine, Qingdao University, Qingdao, China; 3 Department of Clinical Laboratory, the Affiliated Hospital of Qingdao University, Qingdao, China; 4 Qingdao Medical College, Qingdao University, Qingdao, China; 5 Department of Pharmacy, College of Chemical Engineering, Qingdao University of Science and Technology, Qingdao, China

**Keywords:** Food intake, GABA, Nucleus accumbens, Obesity, Orexin-A

## Abstract

**Objective(s):**

To investigate the regulatory effects of the nucleus accumbens (NAcSh)-lateral hypothalamus (LHA) GABAergic neural pathway on palatable food (PF) intake via orexin-A expression in diet-induced obesity (DIO) rats.

**Materials and Methods:**

NAcSh-LHA GABAergic pathways were observed by fluorogold retrograde tracing combined with fluorescence immunohistochemistry, and the regulatory effects of this neural pathway on PF intake were detected after 1) microinjection of GABA-A receptor agonist muscimol (MUS) or antagonist bicuculine (BIC) into LHA, 2) electrical stimulation NAcSh, and 3) blocking the orexin-A receptor by icv SB334867.

**Results:**

Compared with rats on a normal diet (ND), NAcSh-LHA GABAergic neurons in the DIO rats were significantly decreased, and orexin-A expression in LHA significantly increased (*P*<0.05). Microinjection of MUS into LHA significantly decreased the PF intake in both ND and DIO rats (*P*<0.05), and BIC could markedly increase the PF intake in the ND rats (*P*<0.05), but not the DIO rats (*P*>0.05). After NAcSh electrical stimulation or SB334867 ICV injection, the PF intake was significantly decreased in the DIO rats (*P*<0.05), and there was no significant difference after preadministration of BIC into LHA (*P*>0.05).

**Conclusion:**

This GABAergic pathway could regulate the expression of orexin-A in LHA and PF intake. Orexin-A neurons in LHA of DIO rats might be less sensitive to GABAergic signals and may consequently lead to more hedonic food intake.

## Introduction

According to epidemiological investigations, the overweight condition and obesity have increased rapidly over the past 50 years, which greatly increases the risk of many diseases such as type 2 diabetes, hypertension, myocardial ischaemia, and so on (1). Obese people are usually likelier to ingest food, which is related to the change of reward system and is called “hedonic feeding” (2). Moreover, recent evidence suggests that excessive energy intake caused by “hedonic feeding” is one of the key factors of the overweight condition and obesity (3).

“Hedonic feeding” is believed to be regulated by reward-related brain regions, especially the shell of the nucleus accumbens (NAcSh) (3, 4). This effect could be reversed by lesions of the lateral hypothalamic area (LHA), a key nucleus-regulating feeding behavior (5). In addition, the neural projections from NAcSh to LHA have been verified, including theγ-aminobutyric acid (GABA) signal pathway (6-9). However, it is still unclear whether the GABAergic pathway from NAcSh to LHA plays an important role in the regulation of “hedonic feeding” and obesity.

Orexin-A is a type of orexinergic neuropeptide expressed in LHA, and the number of orexin-A immuno-positive neurons of LHA increases in high-fat diets (10). Moreover, orexin-A has been proven to be involved in the regulation of eating addiction and PF intake to promote obesity (11). Therefore, it is hypothesised that reward-related NAcSh regulates hedonic feeding via orexin-A expression in LHA. 

To verify this assumption, the endogenous orexin-A expressions in LHA of the diet-induced obesity (DIO)

rats after PF intake were observed by immunofluro-chemistry. Then, the adaptive changes of the GABAergic neural pathway from NAcSh to LHA in the DIO rats were explored. Furthermore, the PF intake was recorded after administration of the agonist or the antagonist of the GABA-A receptor in LHA, after electrical stimulation of the NAc and after administration of orexin-A receptor antagonist SB334867 in the lateral ventricles to test the functions of this neural pathway. This study evaluated the roles of NAcSh-LHA GABAergic neural pathway, with a focus on downstream orexin-A signaling, to provide a potential novel treatment strategy for obesity.

## Materials and Methods

***Animals***

In all, 192 male Wistar rats were included in the study (weight 180~200 g, animal protocol number: SCXK (Lu) 20190003). The experimental animals were fed at a temperature of 22 ± 2 °C, a relative humidity of 58 ± 5%, and a light/dark cycle of 12 hr (8:00 am‒8:00 pm). Protocols were approved by the Qingdao University Animal Care and Use Committee.

***Experimental instruments and reagents***

The stereotaxic instrument was obtained from Scientifica (UK). Rabbit anti-GABA primary antibody, sheep anti-orexin-A primary antibody, rabbit anti-nesfatin-1 primary antibody, rabbit anti-GABA receptor and primary antibody were purchased from Abcam (UK). Sheep anti-rabbit Cy3 secondary antibody, donkey anti-rabbit Cy3 secondary antibody, and donkey anti-sheep FITC secondary antibody were purchased from Jackson ImmunoResearch (USA). GABA-A receptor agonist muscimol (MUS), GABA-A receptor antagonist bicuculline (BIC) and orexin-A receptor antagonist SB334867 were purchased from Sigma (USA). High-fat diet (# D12492, 5.24 kcal/gm, containing 20% protein, 20% carbohydrates, 60% fat) was obtained from Qingdao Haizhida Biotechnology Co. (China). Sweetened condensed milk (SCM, consisting of 22% fat, 67% sugar, and 10% protein; 3.25 kcal/g and diluted 1:2 in tap water) was purchased from Nestle (Australia).

***Experimental methods***

*Experimental design*

Experiment 1: Twenty Rats were randomly divided into two groups: One group was given a normal diet (ND), and the other group was given a high-fat diet. Body weight was measured weekly. Eight weeks later, 6 ND rats and 6 DIO rats were randomly selected to observe their PF intake. 

Experiment 2: Six ND rats and 6 DIO rats were randomly selected to observe expression of orexin-A and nesfatin-1 in LHA after PF intake by fluorescence immunohistochemistry.

Experiment 3: Six ND rats and 6 DIO rats were randomly selected to observe the coexistence of fluorogold and GABA immunoreactive neurons in the NAc and the co-expressions of orexin-A and GABA-A receptors in LHA by retrograde tracing combined with immunofluorescence histochemical staining. 

Experiment 4: Twenty-four ND rats and 24 DIO rats were randomly divided into three groups. The PF intake was monitored after normal saline (NS), GABA-A receptor agonist MUS, or GABA-A receptor antagonist BIC were microinjected into LHA in ND rats and DIO rats, respectively. 

Experiment 5: Thirty-two ND rats and 32 DIO rats were randomly selected and divided into four groups: (a) NS + sham stimulation group (SS); (b) NS + electrical stimulation group (ES); (c) BIC + SS; (d) BIC + ES. The palatable food intake in the ND and DIO rats was observed after the GABA-A receptor blocker BIC was injected into LHA, and NAc was stimulated by electrical stimulation.

Experiment 6: Twenty-four ND rats and 24 DIO rats were randomly divided into three groups, (1) NS + NS, (2) SB334867 (SB) + NS, and (3) SB + BIC, to observe the effect of SB334847 i.c.v injection, an orexin-A receptor antagonist, on palatable food intake before administration of BIC in LHA of the ND and DIO rats.

*Diet-induced obesity rat model*

After 7 days of adaptive feeding, the rats were fed a high-fat diet, and their body weight was measured every week. After 8 weeks, the rats whose body mass exceeded 20% of the ND rats were defined as DIO rats. The remaining rats were fed normal chow pellets, and all rats drank tap water *ad libitum*.

*Palatable food intake*

Before the experiment, the rats were raised alone, feeding and drinking freely. At 9:00 pm on the day of the experiment, SCM was provided to rats in the ND group and DIO group, respectively; the intake of SCM was recorded for 30 min/d. The method for determining the palatable food intake included weighing the SCM before and after the experiment and calculating the consumption on the 2^nd^, 4^th^, 6^th^, and 8^th^ days to determine the SCM intake of rats.

*Fluorescence immunohistochemistry*

Rats were anaesthetised by thiobarbitol (100 mg/kg, IP) and were perfused and fixed with 4% paraformaldehyde solution. The brains were dehydrated in 30% sucrose and then sliced into 20 μm thick coronal sections. Brain sections were blocked with sheep or donkey serum at room temperature for 2 hr. The sections were incubated overnight at 4 °C with primary antibody (sheep anti-orexin-A 1: 400/rabbit anti-nesfatin-1 antibody 1: 200/rabbit anti-GABA-A receptor 1: 300 /rabbit anti-GABA-A antibody 1:500). Later, the sections were washed three times with PBS and incubated with fluorescent-labelled secondary antibody (Cy3 1:300/FITC1:50) at room temperature for 2 hr, then washed again with PBS three times. Finally, the slices were sealed with anti-quenching sealant and observed with a fluorescence microscope. Using an image analysis system (Jetta Technology Co., Nanjing, China), five fields of five brain slices of LHA or NAc of each rat were counted for immunoreactive cells. The area of cell count was 350 × 350 μm^2^. The percentage of double-labelled cells (%) = the number of double-labelled cells/the total number of positive neurons × 100%.

*Fluorogold retrograde tracing*

After rats were anaesthetised by thiobarbitol (100 mg/kg, IP), they were fixed on the stereotaxic instrument. According to the stereotaxic atalas of Paxinos and Watson (12), 0.2 μl 2% fluorogold (FG) was injected into LHA (Bregma: P:-3.6 mm, L: 1.0 mm and H: 9.6 mm) for retrograde tracing. Penicillin IP (8×10^4 ^U) was provided for 3 days to prevent infection. Seven days later, the rats were perfused with 4% paraformaldehyde solution. Then the brains were removed, and frozen sections were made. The sections were stained by fluorescence immunohistochemistry and the GABAergic projections from NAc to LHA were observed by a fluorescence microscope.

*Drug microinjection*

The rats were fasted overnight and anaesthetised with thiobarbitol (100 mg/kg, IP). Then, they were placed in the stereotaxic frame. A longitudinal incision was made, and the skull was fully exposed. According to the rat brain atlas (12), a 24 G stainless steel cannula was implanted in LHA or the lateral ventricle and fixed with dental acrylic cement. GABA-A receptor agonist MUS (100 ng on each side) or GABA-A receptor blocker BIC (80 ng on each side) in LHA and SB334867 in the lateral ventricle were injected, respectively. Then, 29 G injection cannula and microinjector were connected through a polyethylene tube (10 cm) for drug injection. The rats were intraperitoneally injected with 8×10^4 ^U penicillin for 3 days to prevent infection, and the follow-up experiment was conducted 1 week after the operation.

*Electrical stimulation of the NAc*

A monopolar stimulation electrode was inserted into the NAc (Bregma: P: 2.7 mm, L: 1.4 mm, H: 6.8 mm) and connected with the stimulator. The stimulation parameters were as follows: 20 μA intensity, 0.5 ms duration, which lasted 1 hr at 50 Hz (13). The control group was given sham stimulation (SS), that is, the electrode was implanted, but no electrical stimulation was given.

*Histological verification*

At the end of the experiment, each rat was perfused and fixed, and 50 µm frozen sections of the brain were selected to verify the locations of nuclear injection or electrical stimulation. Incorrectly positioned data were excluded from statistical analysis.

***Statistical processing***

SPSS 17.0 statistical software was used to analyse the data. The experimental results were presented as mean ± SD. Comparisons were made between groups using the Student’s t-test (two groups only), and one-way or two-way ANOVA with *post hoc* Bonferroni test were used for comparisons among means. *P<0.*05 was considered statistically significant.

## Results

***The palatable food intake in DIO and ND rats***

Rats with no significant difference in body weight were randomly divided into ND and DIO groups. After 8 weeks, the body weight of the rats in the DIO group was 440.01 ± 10.86 g, which was 24.73 ± 6.85% higher than that in the ND group (352.71 ± 22.16 g) (*P<0.*05). It indicated that the DIO rat model was established. Within 8 days after administration of PF, the intake of PF in the ND group and DIO group showed an increasing trend. The intake of PF in the DIO group on the 6^th^ and 8^th ^days were significantly higher than in the ND group (*P*<0.05, Table 1). 

***Effect of PF on the expression of LHA neurons in DIO rats***

**Figure 1:**
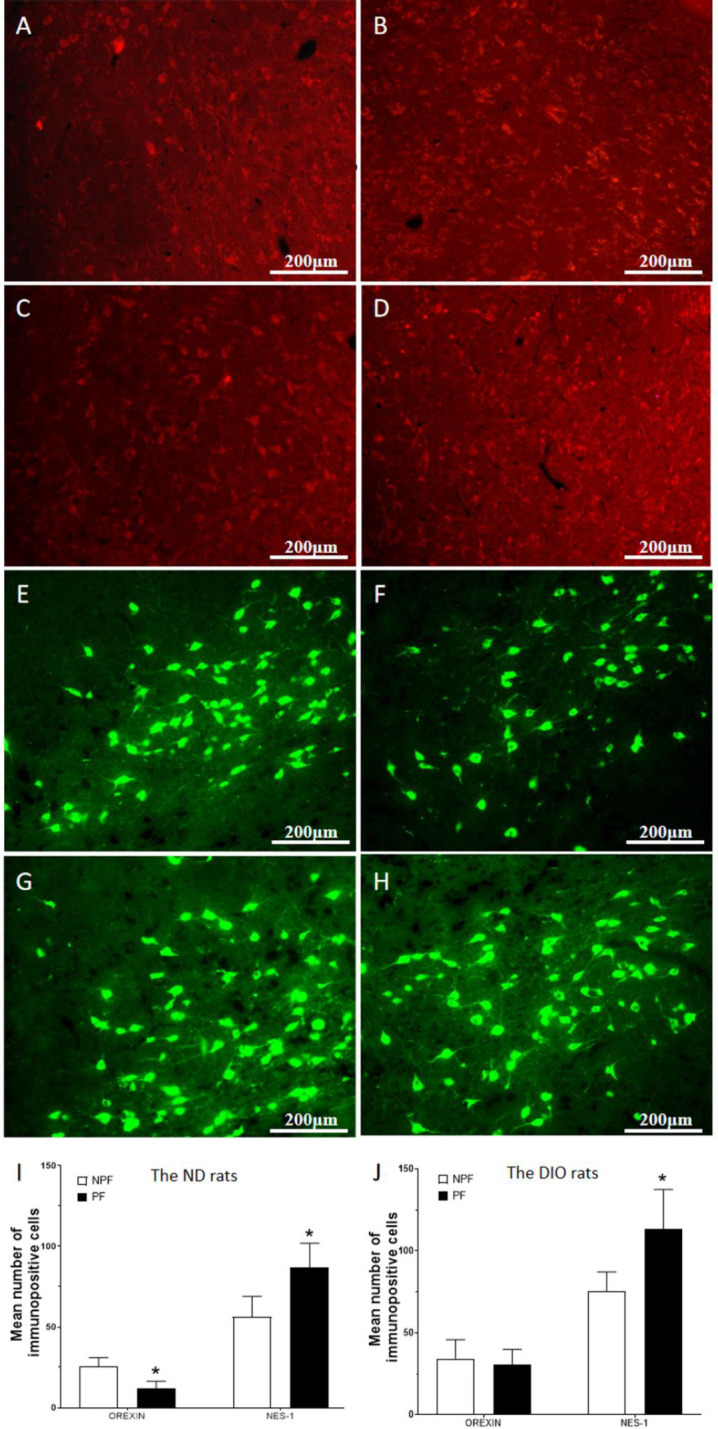
Orexin-A and nesfatin-1 expression in LHA after palatable food intake in the ND and the DIO rats

In this study, immunofluorescence staining was used to observe the difference in the expression of orexinergic neuropeptide orexin-A and anorectic neuropeptide Nesfatin-1 in LHA of DIO rats and ND rats after PF intake. The results showed that after intake of PF, the expression of nesfatin-1 in LHA increased significantly in the ND rats (*P<*0.05, Figures 1A, B, and I) and in the DIO rats (*P*<0.05, Figures 1C, D, and J) , and the expression of orexin-A in LHA of the ND rats significantly decreased (*P*<0.01, Figures 1E, F, and I), while it did not change significantly in the DIO rats (*P*>0*.*05, Figures 1 G, H, and J). 

***Changes in the NAc-LHA GABAergic pathway in the DIO rats***

**Figure 2:**
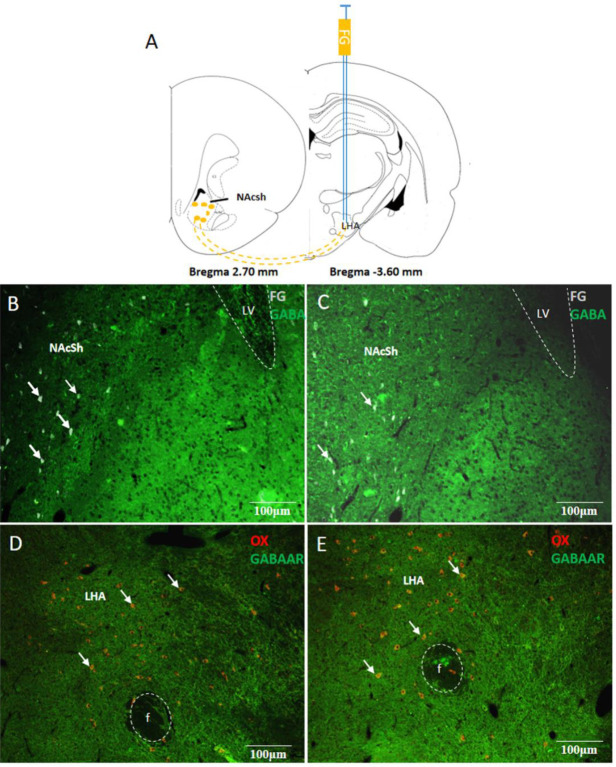
NAcSh-LHA GABAergic projection and co-expression of GABA-AR and orexin-A in LHA

To observe whether theF changes in the orexin-A expression in LHA of DIO rats were due to the activation of the reward closely related nuclei NAc, a retrograde tracer FG was injected into LHA (Figure 2A). Seven days later, FG was found in some neurons in NAcSh, indicating that some neurons in NAcSh projected to LHA. At the same time, immunofluorescence staining was performed to show expression of the inhibitory neurotransmitter GABA in NAcSh. The results showed that GABA and FG coexisted in some neurons of NAcSh in ND and DIO rats. The coexisting neurons in the DIO rats were 8.68 ± 2.34/field, which was significantly lower than that in the ND rats (16.34 ± 5.27/field, *P*<0.05, Figures 2B and C). As seen in Figures 2D and E, there were orexin-A and GABA-A receptor co-expression neurons in LHA of the ND rats and DIO rats, which were 26.75 ±8.15/field and 23.26 ±7.47/field, respectively (*P*>0.05). These results indicated that some GABA neurons in NAc were projected to LHA, and the number of GABA neurons in the DIO rats markedly decreased.

***The effect of microinjection of MUS or BIC into LHA on PF intake in DIO rats***

**Figure 3:**
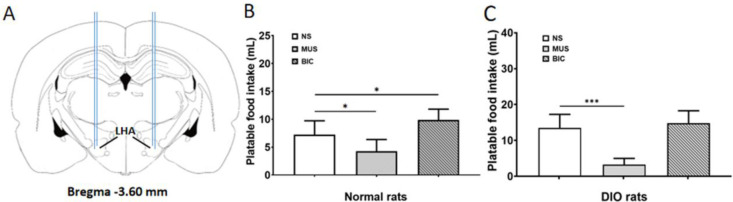
Effects of LHA microinjection of GABA-AR agonist MUS or GABA-AR antagonist BIC on the palatable food intake in the ND and DIO rats

To explore the effect of the GABAergic pathway on the PF intake in the DIO rats, the GABA-A receptor agonist MUS or the antagonist BIC was bilaterally injected into LHA (Figure 3A). Administration of MUS in LHA significantly inhibited the PF intake of the ND rats (*P*<0.05, Figure 3B), and it significantly decreased the PF intake of the DIO rats (*P*<0.001, Figure 3C). BIC significantly increased PF intake of the ND rats (*P<0.*05, Figure 3B), but it had no significant effect on the PF intake of the DIO rats (*P*>0.05, Figure 3C). 

***Effect of NAcSh electrical stimulation on PF intake in DIO rats***

Electrical stimulation of NAcSh was used to observe the effect of the GABAergic signal in NAcSh on the PF intake in the DIO rats (Figure 3D). After electrical stimulation of NAcSh, the PF intake of the NS + ES group in the ND rats was significantly lower than that in the NS + SS group (*P*<0.05, Figure 3E). After preadministration of BIC in LHA, the intake of PF of the BIC + ES group was significantly higher than that of the NS + ES group in ND rats (*P*<0.05), suggesting that BIC could partially block this inhibitory effect of ES, as shown in Figure 3E. Similarly, after the electrical stimulation in NAcSh of DIO rats, the intake of PF was significantly lower than that in the NS+SS group (*P*<0*.*05, Figure 3F). After microinjection of BIC into LHA of the DIO rats, there was no significant difference in PF intake between the BIC + ES group and the NS + ES group (*P*>0.05, Figure 3F). 

***Effect of orexin-A signals blocking on the PF intake in the DIO rats***

**Figure 4:**
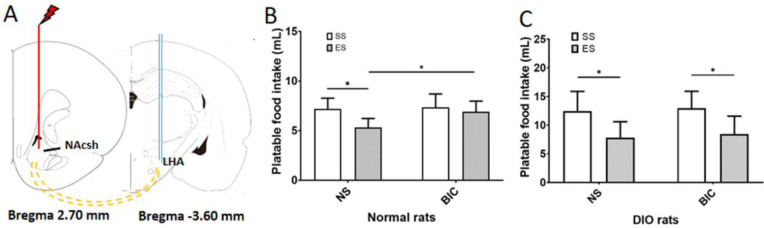
Effects of electrical stimulation of NAcSh on the palatable food intake in the ND and DIO rats

To verify whether orexin-A is the downstream signal of GABA to regulate the PF intake in rats, the effect of ICV injection of orexin-A receptor blocker SB334867 on PF intake was observed (Figure 4A). The intake of PF after ICV injection of SB334867 in the ND and DIO rats was significantly lower than that in the control groups (*P*<0.05, Figures 4B, C). The PF intake of the SB + BIC group in the ND and DIO rats was not significantly different from that of the SB + NS group (*P*>0.05, Figures 4B, C). 

A: Nesfatin-1 immunoreactive neurons in LHA of the ND rats without PF intake; B: Nesfatin-1 immunoreactive neurons in LHA of the ND rats after the PF intake; C: Nesfatin-1 immunoreactive neurons in LHA of the DIO rats without PF intake; D: Nesfatin-1 immunoreactive neurons in LHA of the DIO rats after the PF intake. E: Orexin-A immunoreactive neurons in LHA of the ND rats without the PF intake; F: Orexin-A immunoreactive neurons in LHA of the ND rats after the PF intake; G: Orexin-A immunoreactive neurons in LHA of the DIO rats without the PF intake; H: Orexin-A immunoreactive neurons in LHA of the DIO rats after the PF intake; I: The number of the immunopositive cells in the ND rats. J: The number of immunopositive cells in the DIO rats. scale bar = 200 μm (n = 6)

LHA: lateral hypothalamus; ND: the normal diet; DIO: diet-induced obesity; PF: palatable food

**Table 1:** Palatable food intake in the high-fat diet-induced obesity (DIO) rats (ml)

	2^nd^ day	4^th^ day	6^th^ day	8^th^ day
Normal diet (ND) rats DIO rats	4.02 ± 1.21 6.21±1.93	6.01 ± 1.95 8.29±2.74	6.47 ± 2.13 11.03±3.27^*^	7.21 ± 2.26 12.43±3.39^*^

*P<0.05, compared with the ND rats (n = 6)

A: Sites of FG injection in LHA and retrograde tracing of FG to NAcSh; B: FG and GABA dual-labelled neurons in NAcSh of the ND rats; C: FG and GABA dual-labelled neurons in NAcSh of the DIO rats; D: GABA-AR and orexin-A dual-labelled neurons in LHA of the ND rats. E: GABA-AR and orexin-A dual-labelled neurons in LHA of the DIO rats. The scale bar of B to E is 100μm (n = 6)

NAcSh: the shell of nucleus accumbens; LHA: lateral hypothalamus; FG: fluorogold; ND: the normal diet; DIO: diet-induced obesity; OX: orexin-A; GABA-AR: GABA-A receptor

A: Sites of microinjection in LHA. B: Effects of LHA microinjection of MUS or BIC on PF intake in the ND rats. C: Effects of LHA microinjection of MUS or BIC on PF intake in the DIO rats. *P<0.05, ***P<0.001 (n = 8)

LHA: lateral hypothalamus; NS: normal saline; ND: normal diet; DIO: diet-induced obesity; PF: palatable food; MUS: muscimol; BIC: bicuculine; GABA-AR: GABA-A receptor

A: Sites of microinjection in LHA and electrical stimulation of the NAc. B: Effects of ES in the NAc on PF intake in the ND rats. C: Effects of ES in the NAc on PF intake in DIO rats, *P<0.05 (n = 8)

NS: normal saline; SS: sham stimulation; ES: electrical stimulation; ND: normal diet; PF: palatable food; MUS: muscimol; BIC: bicuculine

**Figure 5:**
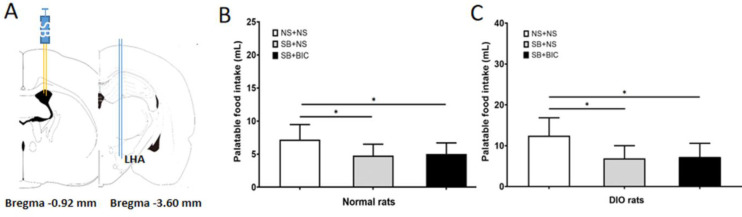
Effects of BIC microinjection in LHA after lateral ventricle injection of SB334867 on the palatable food intake in the ND and DIO rats

A: Sites of microinjection in the lateral ventricle and in LHA; B: Effects of BIC microinjection in LHA after lateral ventricle SB334867 injection on palatable food intake in the ND rats. C: Effects of BIC microinjection in LHA after the lateral ventricle injection of SB334867 on the palatable food intake in the DIO rats, *P<0.05 (n = 8)

NS: normal saline; BIC: bicuculine; SB: SB334867

## Discussion

In this study, it was found that DIO rats had higher orexin-A expression in LHA and lesser GABAergic projection from NAcSh to LHA after PF intake. The change in the GABAergic pathway in the DIO rats influenced PF intake, and the orexin-A receptor antagonist could significantly decrease the PF intake in DIO rats.

LHA is a key nucleus in the central regulation of food intake, which affects feeding behavior by secreting different neurotransmitters (13). Orexin-A and nesfatin-1 are orexigenic and anorexigenic neuropeptides, respectively, and both are expressed in LHA to regulate food intake (14). LHA, the main hypothalamic feeding regulation center, is a hub that can integrate the afferent signals of the arcuate nucleus, hippocampus, ventral tegmental area, and NAc to regulate feeding behavior (7). Some evidence has suggested that the orexigenic neuropeptide decreased and the anorexigenic neuropeptide increases after the palatable food intake (15), and that the expression of orexin-A in DIO rats was related to the different afferent signals (16). Our results showed that in the DIO rats, the expression of nesfatin-1 in LHA still significantly increased after PF intake, but the expression of orexin-A in LHA did not decrease as in the ND rats. It implied that in the DIO rats, the inhibitory effect of the PF food on the orexigenic neuropeptide was reduced, so that orexin-A was sustained at a relatively high expression level. 

NAc is an important part of the mesencephalic limbic system and plays a key role in the reward of drug abuse and the regulation of natural rewards, such as food and sexual behavior (17, 18). NAc consists of the shell and the core, and accumulating evidence has implicated NAcSh in the control of reward- or drug-seeking behavior by spatial/contextual information (19). GABA is the main inhibitory neurotransmitter in NAcSh; the excitability of GABAergic neurons changes adaptively during rewards and addiction (20), and GABA plays an important role in emotion, cognition, behavior and so on (21). It was reported that there were many GABAergic neurons in the NAc, and LHA could regulate food intake after receiving GABA signals (22). 

We observed the NAc-LHA GABAergic pathway using fluorogold retrograde tracing. The results showed that FG and GABA coexisted in some neurons of NAcSh, which proved that GABA neurons in NAcSh could project to LHA. In addition, the number of GABA and FG double-labelled neurons in the NAc of the DIO rats was significantly less than that in the ND rats, indicating that the number of GABAergic neurons in the NAc projecting LHA decreased in the DIO rats. 

Next, the effects of microinjection of GABA-A receptor agonist MUS or antagonist BIC into LHA were explored, and the results suggested that the PF intake decreased significantly in the DIO rats when MUS was administrated, while it did not change as in the ND rats when BIC was used. It might be related to the decrease of GABAergic inhibitory signals in LHA of the DIO rats. The negative regulation of downstream neurons was weakened, and this might lead to the feeding signal orexin-A expression maintaining high expression levels. The orexin-A and GABA-A receptor coexist in some neurons of LHA in the ND rats and DIO rats, which suggested that the NAc-LHA GABAergic pathway might be involved in the regulation of orexin-A expression in LHA. Paul J. Kenny found that LHA damage could attenuate changes in food intake induced by stimulation of NAc, and the decrease in NAc activity improved the activity of LHA neurons (23), which was consistent with our results.

Furthermore, NAcSh electrical stimulation could affect the orexinergic peptide in LHA through the GABAergic signal pathway to suppress PF intake in the ND rats, while the inhibitory effect of GABA was reduced in the DIO rats.

Based on the sustained high levels of orexin-A in LHA of the DIO rats, i.c.v. injection of SB334867 significantly decreased PF intake, and regardless of whether GABA-A receptor blockers were preadministrated in LHA, there was no significant effect on PF intake in rats. The adaptive changes of NAcSh to PF in the DIO rats can promote the intake of PF and increase energy intake by reducing the inhibitory effects on orexin-A expression in LHA.

## Conclusion

Our results demonstrated that GABA neurons in NAcSh participated in inhibitory effects on LHA. The signals of GABAergic neurons from the NAc to LHA decreased in obesity, resulting in a relative increase of orexigenic peptide in LHA and promoting the PF intake. The current study helped clarify the neural pathway from GABA neurons in NAcSh to the orexin-A neurons in LHA and also enriched the experimental basis of “hedonic feeding” and obesity.

## Authors’ Contributions

SG, FG study conception and design; JK, XH, NZ, JH, PJ, SY, XR, YW data processing, collection, perform experiment; SG, FG analysis and interpretation of results; JK, SG draft manuscript preparation, visualization; YG, FG critical revision or editing of the article; SG, FG Final approval of the version to be published; SG, FG supervision, funding acquisition. JK and SG were considered co-first authors.

## Conflicts of Interest

The authors declare that no conflict of interest exists.
